# Automation of a Capillary‐Wave Microbioreactor Platform to Enhance Phage Sensitivity Screen Efficiency

**DOI:** 10.1002/elsc.70021

**Published:** 2025-04-14

**Authors:** Kevin Viebrock, Ilka Knoke, Leon Huß, Detlev Rasch, Sven Meinen, Andreas Dietzel, Rainer Krull

**Affiliations:** ^1^ Institute of Biochemical Engineering Technische Universität Braunschweig Braunschweig Germany; ^2^ Center of Pharmaceutical Engineering Technische Universität Braunschweig Braunschweig Germany; ^3^ Institute of Microtechnology Technische Universität Braunschweig Braunschweig Germany

**Keywords:** automation, microbioreactors, parallelization, phagograms, nanodispenser

## Abstract

To increase their throughput, reduce laboratory work and improve reproducibility, automation of bioprocesses is gaining in importance nowadays. This applies in particular to microbioreactors (MBRs), which can be easily integrated in highly parallelized and automated platforms and, therefore, be applied for screenings, cell‐based assays, and bioprocess development. One promising pharmaceutical application for MBRs is the performance of phage sensitivity tests called phagograms in phage therapy. However, there is no automated and parallelized platform available so far that fulfills the requirements of phagograms. Therefore, a novel highly parallelizable capillary‐wave microbioreactor (cwMBR) with a volume of 7 µL, which has already been successfully applied for phagograms, was extended by an in‐house built platform for automated fluid addition in the single‐digit nanoliter range. The cwMBR has a phage‐repellent hydrophilic glass surface. Furthermore, a custom‐made highly parallelizable device for biomass measurement in the lower microliter scale was developed and validated in the cwMBR. To prove the applicability of the platform for the generation of phagograms, a phagogram using *Escherichia coli* and automated phage addition was performed. The results indicate a clear lysis of the bacteria by the phages and thus confirm the applicability of performing automated phagograms in the highly parallelizable cwMBR platform.

**Abbreviations**
ANOVA: analysis of variance
CAD: computer‐aided design
cwMBR: capillary‐wave microbioreactor
DO: dissolved oxygen
k_L_a: volumetric mass transfer coefficient
LHS: liquid handling system
MBR: microbioreactor
MOI: multiplicity of infection
MTP: microtiter plate
NLI: normalized light intensity
NTC: negative temperature coefficient thermistor
OD: optical density
PSLD: parallelized spectrometric light detector

## 1 Introduction

Automated miniaturized bioreactors with liquid handling systems (LHS) are gaining in significance nowadays as they can reduce the amount of manual laboratory work significantly and, therefore, facilitate high throughput and unsupervised experiments. These systems enable the implementation of various biotechnologically relevant tasks including screening of bacterial strains or cell cultures, bioprocess development with a fed‐batch option and pH regulation, or automated cell‐based assays [1, 2, 3, 4, 5, 6, 7]. Automated liquid addition is generally feasible by microfluidic pumps or LHS, such as pipetting robots. Thereby, robots are characterized by a simple change of the type of fluid added to the cultivation. However, these systems can only add fluid intermittently, which is disadvantageous, for example, for a continuous feed supply. In contrast, microfluidic pumps can fill fluids continuously leading to lower fluctuations in process parameters like substrate concentration, filling volume, or pH value [8, 9]. Microfluidic pumps, as well as modern LHS, can add fluid volumes down to a nanoliter‐scale nowadays, allowing precise addition of small amounts of, for example, an assay substance to a cultivation [10, 11]. Automated cultivations in miniaturized bioreactors require the implementation of online sensors to monitor the cultivation. Especially in microbioreactors (MBRs) with a volume below 1 mL, optical chemical sensors can perform this task. These sensors enable the non‐invasive measurement of important process parameters like pH value, dissolved oxygen (DO), or glucose concentration [12, 13]. Furthermore, biomass growth can be determined, for example, by impedance, light absorption, or scattered light measurement [2, 13–15].

**Summary**

The rise of multidrug‐resistant bacteria, driven by excessive antibiotic use, poses a significant threat to human and animal health. These resistant strains can cause severe infections. Phage therapy offers a potential solution, utilizing phages that infect and destroy specific bacterial cells, including those resistant to antibiotics. This targeted approach allows phages to effectively combat infections when traditional antibiotics fail. Before treatment, phagograms are necessary to determine bacterial susceptibility to various phages. This requires screening extensive phage libraries, a process currently labor‐intensive and time‐consuming when using double agar overlay plaque assays. However, employing microbioreactors (reaction volumes under 1000 µL), like the capillary‐wave microbioreactor described here, can provide a faster, automated, and cost‐effective method for conducting high‐throughput phagograms, thus streamlining the process and enhancing the potential for phage therapy in addressing antibiotic‐resistant infections.

Different automatable, miniaturized bioreactors are already commercially available including the RoboLector (m2p‐labs/Beckman Coulter), Ambr 15 (Sartorius), micro‐Matrix (Applikon/Getinge), and bioREACTOR 48 (2 mag). However, all of these systems suffer from certain limitations including high parallelization or a relatively large volume of around or even far above 1 mL and parallelization up to 48 reaction chambers. A small volume can increase the throughput and reduce the costs for expensive media components and testing substances [3]. All mentioned commercially available miniaturized bioreactors are applicable for microbial cultivations and are the most common miniaturized bioreactors used in research nowadays [16, 17, 18, 19, 20, 21]. However, these devices suffer from certain limitations. These include a relatively high volume in the milliliter scale, which increases the space requirements and, therefore, reduces the ability to high parallelization. Therefore, all described devices are limited to 48 or less parallel cultivations. Furthermore, the higher media and testing substance consumption increases cultivation costs especially for expensive mammalian cell culture media [3]. Moreover, MTP‐based platforms with a lower volume, in particular, often feature temperature gradients of up to a few degrees Celsius between circumferential and centrally located wells due to inhomogeneous heating, which is called the edge effect. Besides temperature effects on cell growth, these effects lead to evaporation deviations between the wells and, therefore also, concentration deviations. Furthermore, the heating time of unpreheated fluids in an MTP is often high because of the poor heat transfer of the applied plastic materials [22, 23, 24].

**FIGURE 1:**
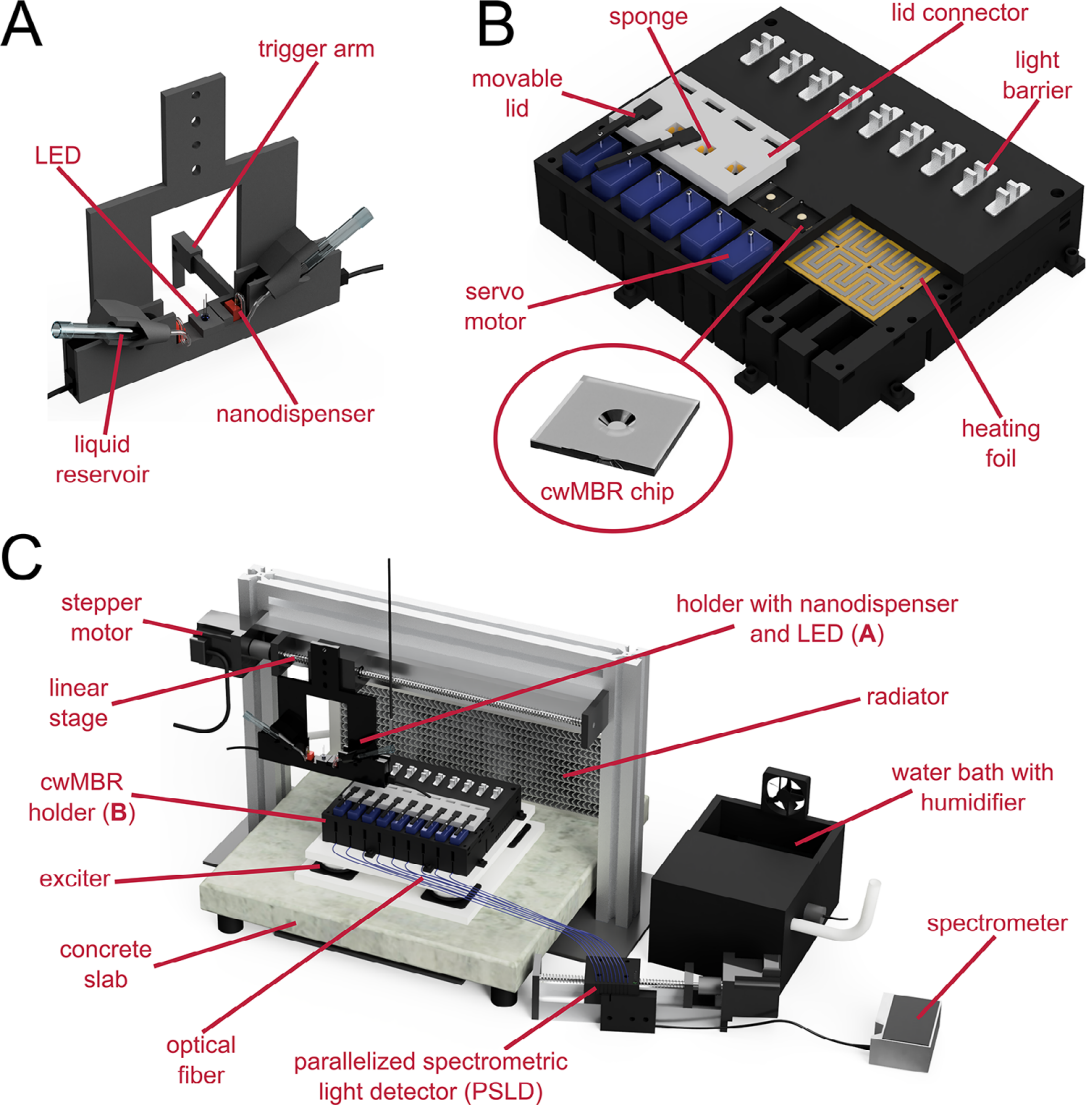
Automated cwMBR platform. (A) 3D‐printed nanodispenser and LED holder. Two nanodispensers are held in position by the holder. Each nanodispenser has a pipette tip as a liquid reservoir connected to the nanodispenser by tubing. The LED is positioned between the two nanodispensers with a distance equal to the distance between two cwMBR chips. A trigger arm can interrupt the light signal of light barriers to control positioning of the holder. (B) 3D‐printed cwMBR holder with cwMBR chips, heating foil, light barriers, and servo‐controlled movable lids. The holder can hold up to nine linearly aligned cwMBR chips. Movable lids with sponge equipped connectors minimize evaporation from the droplet. Fast heating and homogenous temperature distribution are achieved by the heating foil, which is positioned directly under the cwMBR chips. Light barriers control the positioning of the nanodispenser and LED holder. (C) Overview of the entire automated cwMBR platform. The 3D‐printed cwMBR holder is placed on an exciter platform for mixing via vertical oscillation. The nanodispenser is connected to a linear stage with a stepper motor for precise movement over the cwMBR chips. Measurement of the transmitted light intensity is performed by a parallelized spectrometric light detector (see Section 3.3) connected to the cwMBR chips via optical fibers. All devices are placed in an incubation chamber heated via a radiator and thermostat and humidified with an ultrasonic humidifier in a water bath.

One way to circumvent these limitations is to perform the cultivation in sessile droplets in the lower microliter scale, often done on automated droplet microarrays. Therefore, each droplet of a highly parallelized platform acts as one MBR and can be analyzed using, for example, optical sensor tools [25]. The so‐called capillary‐wave microbioreactor (cwMBR) is an MBR, which bundles the advantages of both sides: small volume and, therefore, high parallelizability, integrated optical sensors, and active mixing. The cwMBR mainly consists of a square glass chip (Figure 1B) holding a sessile droplet of cultivation medium with a volume of 7 µL. The droplet is placed in a centrally located cavity. The chip is fabricated using an ablation process called femtosecond laser direct writing in photosensitive Foturan glass [26]. Afterwards, the cwMBR chip is placed in a 3D‐printed mounting for fixation of the cwMBR chip with the cultivation medium. Furthermore, the mounting minimizes evaporation from the cwMBR by creating a small headspace over the droplet, which is humidified by moistened sponges. In contrast to many other droplet‐based MBRs, the cwMBR can be mixed actively by vertical oscillation. Therefore, the cwMBR mounting is affixed to an oscillation platform, which can oscillate the 3D‐printed mounting, including the cwMBR chip, with adjustable frequencies and amplitudes. By oscillation at resonance frequencies of the droplet, capillary waves on the droplet's surface with specific wave patterns are formed. These capillary waves form micro vortices within the droplet, which mix the cultivation media. With this mixing technique, adjustable *k*_L_ *a* values of more than 340 h^−1^ and mixing times below 2 s can be achieved by adjusting the resonance frequencies of the droplet [15, 27]. For process characterization, optical‐chemical sensors for pH, DO, and glucose can be integrated. Furthermore, biomass concentration can be measured using absorbance measurements [28]. Therefore, an LED is placed over the cwMBR chip and emits light through the droplet. As light is absorbed by the biomass in the droplet, transmitted light measurement by a spectrometer or photoresistors as sensors can be used for biomass determination.

So far, the cwMBR has been successfully applied for bacteriophage sensitivity screens called phagograms [29]. Phagograms are essential screens for the application of phage therapy, posing a promising alternative to treat multidrug‐resistant pathogen bacteria. Phage therapy uses bacteriophages, which are highly selective viruses, mostly killing a limited number of bacterial strains. Due to the high selectivity, phagograms are essential for finding an effective phage or phage combination from a large library and, therefore, represent a specific type of personalized medicine. Phagograms are mostly performed before phage therapy. During a phagogram, the infectious bacterium isolated from the patient is cultivated in vitro and various phages from a phage library are added. If a phage is capable of infecting the bacterium, this can be observed by bacterial lysis. Nowadays, phagograms are mainly performed by work‐intensive and less parallelizable and automatable plaque assays, where bacterial lysis through phages is observed by the formation of plaques in biofilms on double‐layer agar plates. As an alternative, phagograms can be performed by cultivating the bacteria with phages in suspension and observing bacterial lysis through a decrease in optical density (OD) [30, 31, 32, 33, 34]. Therefore, highly parallelizable and automatable MBRs like the cwMBR pose a promising alternative to increase the throughput and minimize manual laboratory work for phagograms in future. Due to their hydrophobic and, therefore phage, adsorbing plastic surface, most conventional miniaturized bioreactors and MTPs are not suited for phagograms, which makes the cwMBR with its phage‐repellent hydrophilic glass surface a perfect solution [35].

The aim of this work is to demonstrate the automation of the existing cwMBR platform using a simple and cost‐efficient approach, which can be transferred to comparable platforms. Therefore, a nanodispenser capable of dispensing droplet volumes down to 2 nL was connected to an in‐house manufactured automation platform for nine parallelized cwMBRs. Furthermore, the platform was optimized regarding homogenous temperature distribution and a highly parallelizable absorbance sensor system was introduced. The developed system was applied for automated phagograms using a model system based on *Escherichia coli* K12 and different concentrations of phage vB_EcoM_MM02 (Myovirus).

## 2 Material and Methods

### 2.1 cwMBR Platform With Automated Fluid Addition and Parallelized Biomass Measurement

To compensate for evaporation from the cwMBR and to automatically add phages in phagograms, an automated nanodispenser was integrated in the cwMBR platform (Figure 1). Therefore, the previously described cwMBR platform was used as a basis [15]. The cultivation is performed in a 7 µL droplet positioned in a frustum‐shaped cavity in a Foturan glass chip (Schott, Mainz, Germany). The glass chips are manufactured by femtosecond laser direct writing, as described previously [26]. They were placed in a 3D‐printed holder made of black polylactic acid (Flashforge, Waldshut‐Tiengen, Germany) in a fused deposition modeling printer (i3 MK3S+, Prusa Research, Prague, Czech Republic). To minimize evaporation, the cwMBR is covered by 3D‐printed movable lids and sponge equipped connectors between the lid and holder. The connectors are held in position by round 5 × 1 mm neodymium magnets (Hangyunkejia, Shenzen, China) and sealed with silicone mats. The movable lids can be opened by a servo‐motor (Miuzei Micro Servo 9 g MS18, Prolinx, Düsseldorf, Germany). Light barriers (TCST2103, Temic Telefunken microelectronic, Heilbronn, Germany) were used to control the positioning of the automated nanodispenser. For temperature control in the cwMBR chips, a heating foil (Polyimide heater 45 mm × 155 mm, Thermo Technologies, Rohrbach, Germany) was integrated into the 3D‐printed mounting. The heating foil is connected to a temperature controller equipped with a 10 kΩ negative temperature coefficient (NTC) thermistor (Fox‐301A (controller + NTC), Daesung Engineering, Hwaseong, Republic of Korea). The NTC is glued in the 3D‐printed mounting between two centrally located cwMBR chips to ensure precise temperature determination. The offset between the temperature measured by the NTC and the actual temperature in the cwMBR chips is determined with the help of the optical temperature sensors (see Section 2.2).

For vertical oscillation, the cwMBR holder is mounted on an exciter platform consisting of two polyvinyl chloride boards and four electronic exciters (Ex 45 S, Visaton, Haan, Germany). An oscilloscope software (Soundcard Scope, Essen, Germany) generates an audio signal with the resonance frequency of the droplet (70 Hz), which is transmitted to the exciters by a soundcard (Gigaport HD+, ESI Audiotechnik, Leonberg, Germany) and amplifiers (M034N, Kemo Electronic, Geestland, Germany). Mixing is deactivated for 10 s during absorbance measurement and fluid addition by interrupting the power supply of the amplifiers with a relay (KY‐019RM, Joy‐IT, Neukirchen‐Vluyn, Germany). Due to the short interruption of the mixing and the high oxygen saturation during an *E. coli* cultivation in the cwMBR [28], no oxygen limitations are expected from the deactivation of mixing. To minimize transmission of the vibration to surrounding components, the oscillation platform is glued on a concrete slab (40 cm × 40 cm × 4 cm, Diephaus, Vechta, Germany) with four rubber feet.

Two nanodispensers (PipeJet Nanodispenser, BioFluidix, Freiburg im Breisgau, Germany) are mounted in a 3D‐printed holder connected to a stepper motor controlled linear stage (350mm‐NEMA23, Befenybay, China) for accurate positioning of the nanodispensers. The linear stage is mounted on a 505 mm × 350 mm framing made out of T‐slot aluminum profiles. The one‐dimensional movement of the nanodispenser is operated by a microcontroller (Mega 2560 (Rev3), Arduino SRL, Torino, Italy) connected to the stepper motor via a digital stepper driver (Stepperonline DM542T, OMC, Nanjing, China). A conventional blue 3 mm LED with a wavelength of 465 nm (*I*_V_ = 5 cd) is positioned between both nanodispensers as a light source for absorbance measurement. The microcontroller also controls further integrated devices including the LED, relays, servo motors, stepper motors, and nanodispensers.

The transmitted light intensity is measured using an in‐house built parallelized spectrometric light detector (PSLD, Figure 4A) connected to the cwMBR via optical fibers with a diameter of 400 µm (FT400UMT, Thorlabs, Newton, NJ, USA). The PSLD consists of a second stepper motor controlled linear stage (100mm‐NEMA17, Befenybay, China) moving the optical fibers over a bigger optical fiber with a diameter of 1 mm (FT1000UMT, Thorlabs, Newton, NJ, USA) connected to a spectrometer (Flame, Ocean Insight, Orlando, FL, USA) as a sensor. The measured absorbance is displayed online on a computer by an in‐house programmed Python software (see Supporting Information).

For constant ambient air conditions, the automated cwMBR platform with PSLD is placed in an incubation chamber made out of T‐slot profiles and black polyvinyl chloride plates to protect the sensor from ambient light. The chamber is equipped with humidity and temperature control. The temperature control consists of a radiator (360 G2 Slim, Magicool, Mönchengladbach, Germany) with fans (F12 PWM PST, Arctic, Braunschweig, Germany) connected to a thermostat (Eco E4, Lauda Dr. R. Wobser, Lauda Könighofen, Germany). The temperature is controlled by adjusting the rotation speed of the fans using a second microcontroller (Uno (Rev3), Arduino SRL, Torino, Italy) connected to a thermometer (DSB18B20, Sertronics, Berlin, Germany) and the fans via switched‐mode power supply (LRS‐35‐12, Mean Well, New Taipei City, Taiwan). The humidity is regulated to 95% by a humidity sensor equipped controller (Fox‐301A, Daesung Engineering, Hwaseong, Republic of Korea) and an ultrasonic humidifier (DH‐24B, Conrad Electronic, Hirschau, Germany) in a 3D‐printed water bath. The Arduino scripts for both microcontrollers can be found in the Supporting Information.

### 2.2 Characterization of the Automated cwMBR Platform and the Parallelized Spectrometric Light Detector

After developing the cwMBR platform, it was characterized regarding liquid level, temperature distribution and accuracy of the absorbance measurement. The cwMBR was equipped with two automated nanodispensers: one for refilling the cwMBR with evaporated water and the other for automated phage addition. To keep the liquid level in the cwMBR constant, the evaporation rate from the automated cwMBR platform was determined and the evaporated volume was refilled every 15 min according to the evaporation rate. Therefore, the fully assembled cwMBR was filled with 7 µL complex medium (see Section 2.3) and incubated under cultivation conditions of 37°C and 95% relative humidity with activated mixing (70 Hz oscillation frequency, 5% amplitude). After specified time intervals (10, 15, 20, 30, 45, 60, and 90 min), the remaining volume was measured in triplicates using a pipette (Eppendorf Research Plus, Eppendorf, Hamburg, Germany), and the evaporation rate was determined. The resulting evaporation rate of 1.84 µL ∙ h^−1^ ± 0.03 µL ∙ h^−1^ is equated by deionized water addition of 0.46 µL every 15 min using the nanodispenser. The measurement error results from the measurement deviation of the pipette. The manufacturer of the nanodispenser specifies a coefficient of variation of 0.37% for a comparable volume of 0.5025 µL^1^. This means that the expected standard deviation of the water addition is 1.7 nL, which ensures a high level of accuracy. To test, if the liquid level is constant over a cultivation period of 8 h, the liquid level from three cwMBR chips was measured hourly using a pipette in a separate experiment with activated water addition under cultivation conditions.

To investigate the temperature distribution in the cwMBR platform and its heating time, eight cwMBR chips were equipped with optical temperature sensor spots (TPSP5‐ADH, Pyroscience, Aachen, Germany). The temperature was measured using a fiber‐optics read‐out device (FireSting‐Pro, Pyroscience, Aachen, Germany) connected to the cwMBR chips via optical fibers (FT400UMT, Thorlabs, Newton, NJ, USA). Calibration was performed in a water bath with a preset temperature of 37°C. The calibration temperature of the water bath was determined using a thermometer with Pt1000 temperature probe (TD20, VWR International, Leuven, Belgium) with an accuracy of 0.1 K. For measurement of the heating performance, the cwMBR chips were filled with 7 µL water at room temperature and incubated under cultivation conditions (37°C, 95% relative humidity, 70 Hz oscillation frequency, 5% amplitude). The temperature was measured every second over 40 min in three iterations. To compare the heating performance to established MTP readers and MBRs, a 96 well plate (96 Well Microplate, F‐Bottom, Chimney Well, Greiner Bio‐One, Kremsmünster, Austria) and a FlowerPlate (m2p labs (Beckman Coulter), Aachen, Germany) were equipped with eight randomly distributed temperature sensor spots for measurement with the fiber‐optics read‐out device. The sensor spots were calibrated in the same water bath used for the cwMBR chips with a preset temperature of 37°C. The 96‐well plate was filled with 200 µL water at room temperature and incubated in a plate reader (Spark, Tecan, Männedorf, Switzerland) at 37°C, a shaking frequency of 1440 min^−1^ and a shaking diameter of 1 mm. The FlowerPlate filled with 1 mL water at room temperature was incubated in a conventional MBR (BioLector XT, m2p labs (Beckman Coulter), Aachen, Germany) at 37°C and a shaking frequency of 1400 min^−1^. The temperature in both reference devices was measured every 10 min by removing the plate from the device for a few seconds.

For parallelized biomass measurement via absorbance, the PSLD was developed (see Section 2.1). To validate the device, it was characterized regarding the repeatability and the linearity of the sensor signal, and the OD in the cwMBR chip. For validation of the linearity, different dilutions of a turbidity standard (Formazin Turbidity Standard 4000 NTU, Hach, Loveland, CO, USA) were prepared with predefined ODs between 1 and 6. Afterwards, 7 µL of the dilutions were filled in the cwMBR chips and the transmitted light intensity was measured three times per cwMBR chip using the PSLD and the blue LED. Mixing (70 Hz oscillation frequency, 5% amplitude) was activated between the measurements to avoid sedimentation. To validate the repeatability of the PSLD signal, the transmitted light intensities of all nine empty cwMBR chips were measured during ten iterations of the PSLD as triplicates. Mixing of the automated cwMBR platform was activated between the measurements (70 Hz oscillation frequency, 5% amplitude).

### 2.3 Phagograms in the Automated cwMBR Platform

To prove the applicability of the automated cwMBR for phagograms, a model phagogram was performed. Therefore, *E. coli* K12 MG1655 (DSM 18039) and the phage vB_EcoM_MM02 (short: MM02; DSM 29475) were purchased from the German Collection of Microorganisms and Cell Cultures (DSMZ, Braunschweig, Germany) and used as bacterium and model phage, respectively. Bacteria and phage preparation were performed as described previously [29]. Therefore, a complex medium consisting of 5 g ∙ L^−1^ yeast extract (Carl Roth, Karlsruhe, Germany), 10 g ∙ L^−1^ peptone from soybean (enzymatic digest, Merck, Darmstadt, Germany), and 10 g ∙ L^−1^ sodium chloride (Merck, Darmstadt, Germany) was used. For inoculation, a preculture of *E. coli* in the complex medium with 8.2 mM magnesium sulfate (Carl Roth, Karlsruhe, Germany) was cultivated in a shake flask with baffles at 37°C, 140 min^−1^ and a shaking diameter of 50 mm (Certomat IS, Sartorius, Göttingen, Germany) until an OD of 0.5 was reached. The cwMBR chips were inoculated with 6.5 µL of the preculture and incubated at 37°C and 95% relative humidity with activated oscillation (70 Hz oscillation frequency, 5% amplitude). After 1 h, a phage solution with a phage titer of 6.25 ∙ 10^7^ mL^−1^ was automatically added by the nanodispenser, resulting in two different multiplicities of infection (MOI) of 10^−3^ and 10^−5^. The MOI describes the ratio of phages to bacteria during phage inoculation and was estimated by the initial cell number, the growth rate, and the concentration of the phages added to the cultivation. The phagogram was performed in triplicates and three cwMBR were used as growth control without phages. Evaporation (1.84 µL ∙ h^−1^) from the cwMBR is equated by automated 0.46 µL deionized water addition every 15 min. To further quantify cell growth, microscopy images of the preculture and the final culture medium were taken. Cell counting was performed using a counting chamber (Neubauer improved chamber, Brand, Wertheim, Germany) and an optical microscope (Axioskop, Carl Zeiss, Oberkochen, Germany) with 40‐fold magnification. The counted cell number was used to calculate the cell number during cultivation as described previously [29]. Therefore, cell number and PSLD sensor signal are proportional.

### 2.4 Statistical Analysis

For analysis of the time, the cwMBR needs to reach an equal temperature compared to the target temperature of 37°C (expected value) during heating experiments, a one‐sample *t*‐test with a significance level of *α* = 0.05 was performed. The same test was performed to control, if the maximal deviation of the liquid level in the cwMBR is equal to the expected value. To test, if there is a significant difference in the final temperature of the automated cwMBR platform, FlowerPlate, and MTP, single‐factor analysis of variance (ANOVA) was used. A *p* value of 0.05 was chosen as the threshold. This means that a calculated *p* value lower than 0.05 represents a statistically significant difference between the final temperature in the different devices.

## 3 Results and Discussion

### 3.1 Automated Fluid Addition in the cwMBR

The aim of this work is to develop a cwMBR platform to perform automated phagograms. Therefore, an existing cwMBR platform is to be further developed and, among others, extended by two nanodispensers for automated fluid addition. Thereby, the nanodispensers have two main tasks: refilling the cwMBR to counteract evaporation and automatically adding phages for phagograms. A CAD image of the developed platform is displayed in Figure 1.

To achieve mixing of the cwMBR, the exciter platform developed by Frey et al. [15] and Meinen et al. [26] was utilized (Figure 1C). This platform vertically oscillates a 3D‐printed cwMBR holder mounted on the platform with four exciters. This vertical oscillation induces capillary waves mixing the cwMBR droplet. To reduce the transmission of the oscillation to surrounding components, the exciter platform was mounted on a concrete slab with flexible rubber feet. The 3D‐printed mounting (Figure 1B) can hold up to nine linearly aligned cwMBR chips and holds them in position for vertical oscillation. Furthermore, the mounting fixates optical fibers, which conduct transmitted light from the OD measurement to the PSLD (see Section 3.3). To minimize evaporation, the cwMBR chips are covered by servo‐controlled movable lids minimizing the headspace over the cwMBR. To further minimize evaporation, the connectors between movable lids and cwMBR are equipped with moistened sponges increasing the humidity within the headspace (Figure 1B). A similar setup was already successfully applied for previous unautomated cwMBR platforms. To achieve a homogenous temperature distribution and fast heating (see Section 3.2), a heating foil was integrated in the 3D‐printed mounting directly under the cwMBR chips (Figure 1B). Light barriers screwed on the mounting are used to control positioning of the nanodispenser in the automated setup.

T‐slot profiles are applied to build a structural framing for the automated nanodispenser (Figure 1C). A linear stage consisting of a spindle axle with a slide and a stepper motor is mounted to the framing for accurate movement of the attached nanodispenser. The nanodispenser is connected to the linear stage via a 3D‐printed holder (Figure 1A). The setup with a spindle axle and stepper motor is mostly applied in precise 3D printers and is, therefore, perfectly suitable for low‐cost, accurate positioning in the automated cwMBR setup. The stepper motor is able to perform exact steps of 1.8° resulting in a reproducible movement of 0.025 mm of the attached nanodispenser. To control the positioning of the nanodispensers, its 3D‐printed holder is equipped with a trigger arm (Figure 1A), which interrupts the signal of the light barriers (Figure 1B) of a corresponding cwMBR chip if the nanodispenser is aligned with the chip. The nanodispensers can precisely add volumes down to 2 nL. A pipette tip is used as a liquid reservoir for the nanodispenser, which can be filled with either water or phage solution (Figure 1A). An LED is fixed between the two nanodispensers and serves as a light source for OD measurements (see Section 3.3). The distance between the LED and the nanodispensers is equal to the distance between two cwMBR chips. Therefore, all three devices can work parallel if aligned. At the same moment, when the OD is measured, both nanodispensers can add fluid to the aligned cwMBR chips. For OD measurement and liquid addition, the servo‐controlled lids of the cwMBR holder are opened (Figure 1B).

All devices are placed in an incubation chamber for constant conditions of the ambient air. Besides heating the cwMBR holder by the heating foil, the incubation chamber is heated by a thermostat connected to a radiator with fans (Figure 1C). Therefore, the temperature is regulated by the rotation speed of the fans. To increase humidity within the incubation chamber and thereby further reduce evaporation from the droplet, a water bath with an ultrasonic humidifier is placed in the incubation chamber (Figure 1C). All devices are controlled by a microcontroller.

**FIGURE 2:**
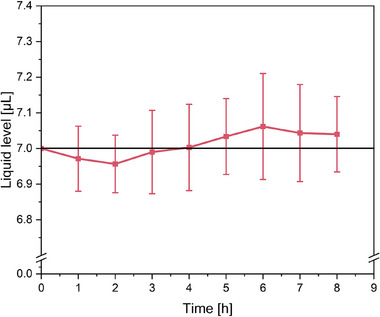
Liquid level in the automated cwMBR during incubation (37°C, 95% relative humidity, oscillation frequency of the cwMBR 70 Hz, amplitude 5%) with automated refilling of deionized water to compensate for evaporation (0.46 µL every 15 min) using the nanodispenser. The initial liquid volume in the cwMBR was 7 µL. The remaining volume was measured after the respective point in time using a pipette. Each value represents the mean and standard deviation from a triplicate.

One nanodispenser is used to refill the cwMBR chips with evaporated water. Therefore, the evaporation rate from the cwMBR chips is determined before the experiment using a pipette. The resulting evaporation rate during cultivation of *E. coli* of 1.84 µL ∙ h^−1^ is equated by water addition every 15 min. To determine if the liquid level is constant over a cultivation period of 8 h, it was measured hourly in a separate experiment over all cwMBR chips. The results are displayed in Figure 2. The results reveal a constant liquid level within the cwMBR platform. The liquid level fluctuates within less than 0.1 µL from the target volume of 7 µL. Even the maximal deviation of 0.062 µL represents only 0.88% of the cwMBR volume. The deviation from the expected volume of 7 µL is not significant with a significance level of *α* = 0.05. Therefore, a constant volume in the cwMBR can be assumed making the automated cwMBR platform perfectly suitable for phagograms with high comparability between the cwMBR chips. Compared to a previous cwMBR setup without automated refilling, the volume deviation after 8 h was reduced from 4.57% to 0.57%, representing a distinct improvement of the cwMBR system. For longer cultivation periods, such as a few days, it would make sense to integrate a liquid level sensor for a full liquid level control. As slight deviations of the replenishment rate from the measured evaporation rate would lead to fluctuating liquid levels in the cwMBR, this effect increases with longer cultivation times resulting in concentration changes during cultivation. The liquid level can be measured, for example, by the reflection angle of a laser beam on the droplet's surface.

### 3.2 Temperature Distribution and Heating Time

**FIGURE 3:**
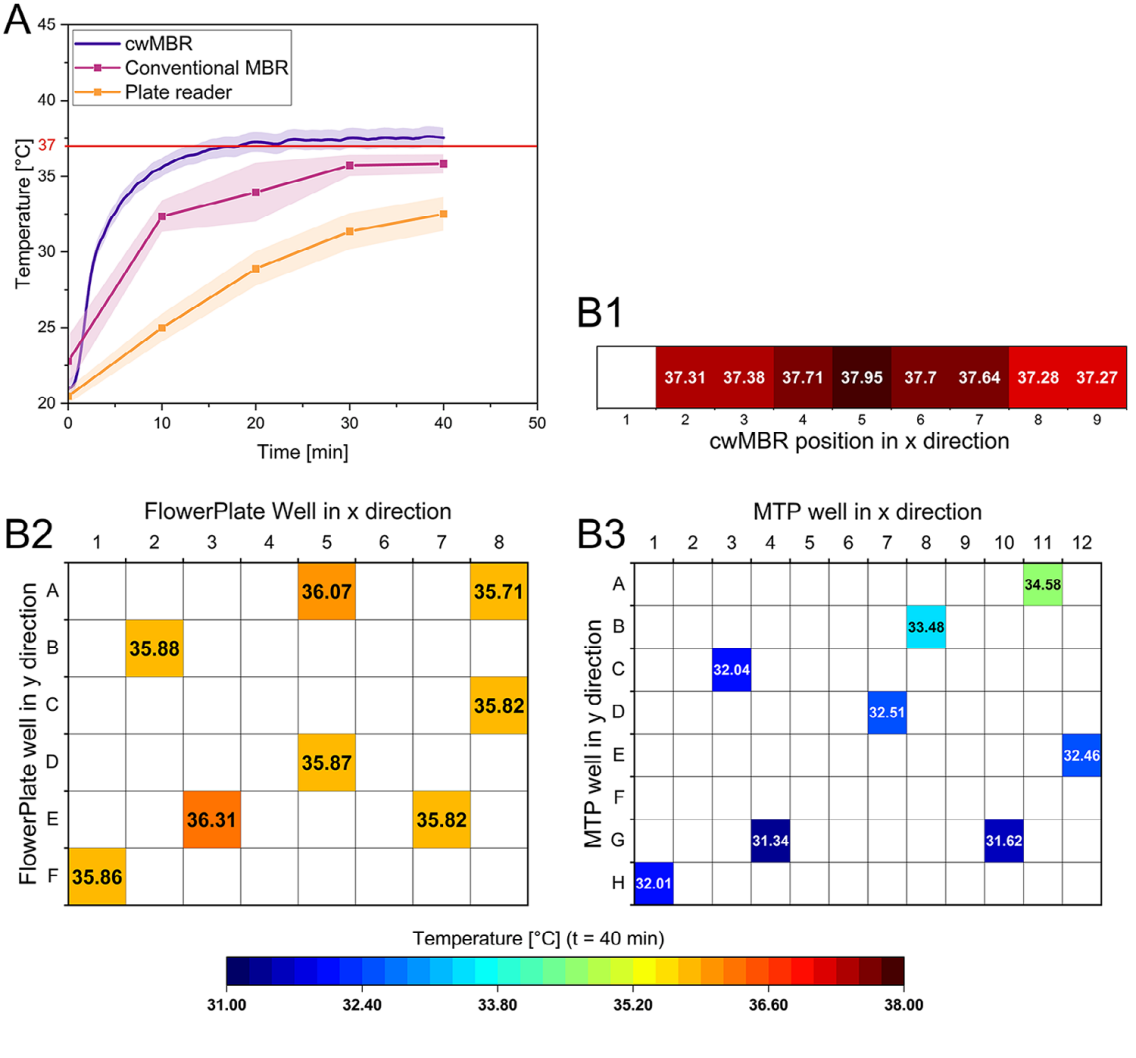
(A) Temperature in the automated cwMBR platform during heating to 37°C compared to a conventional MBR and an MTP reader. The cwMBR was filled with 7 µL water, the conventional MBR with 1000 µL water, and the plate reader with 200 µL water at room temperature. Mixing was activated in all devices during incubation. The temperature was measured using optical sensor spots. Each value represents the mean and standard deviation (lightly colored) from three measurements using eight sensor spots in distributed wells or cwMBR chips. (B) Temperature distribution at the end of the incubation period from (A) over the cwMBR platform (B1), FlowerPlate (conventional MBR, B2), and MTP (B3) from three measurements.

A homogenous temperature distribution, as well as short heating times, are essential for all parallelized cultivation platforms to achieve comparability between the cultivations. Especially in MTP‐based cultivation platforms, temperature deviations between inner and circumferential wells are a major challenge causing different growth kinetics and concentration gradients between the wells by different evaporation rates. This effect is called the edge effect and was investigated by different authors [23, 24, 36]. With classical MTPs and MTP readers, temperature gradients of up to 2.2°C were observed, which have a significant influence on the conducted experiments [22]. Furthermore, due to the low heat transfer through plastic MTPs and the passive heating in MTP readers, heating times are often long [22]. This makes preheating often essential for MTP experiments. In previous publications, the cwMBR was heated passively by the surrounding incubation chamber causing temperature deviations between inner and outer cwMBR chips and increasing the heating time. Therefore, a heating foil was integrated in the cwMBR mounting to decrease heating time and temperature deviations between the cwMBR chips. To evaluate both parameters, the heating time and the temperature distribution were measured in eight cwMBR chips of the automated platform for 40 min and compared to a conventional MBR platform and an established MTP reader (Figure 3).

Figure 3A illustrates the average temperature over the cwMBR chips in a period of 40 min compared to the MTP reader and the conventional MBR as reference devices. The results indicate a significantly reduced heating time of the cwMBR compared to reference devices. After 10 min of heating, the cwMBR had a temperature of 35.5°C and, therefore, almost reached the target temperature of 37°C. This temperature is reached with a significance level of *α* = 0.05 after 14.9 min. At the same time, the temperature in the plate reader (25.0°C) in particular, and the temperature in the conventional MBR (32.4°C) are both far from the target temperature. The cwMBR is the only platform reaching the target temperature within the experiment time, even though the final temperature in the conventional MBR is close to the target. Both reference devices contain larger volumes of 1000 µL (conventional MBR) or 200 µL (MTP) compared to the small volume of 7 µL in the cwMBR, which needs more time for heating. This demonstrates one advantage of small MBR volumes: faster heating times and, therefore, faster experiments. Furthermore, the applied heating foil in the cwMBR directly heats the cwMBR chips, while heating by ambient air through plastic MTPs takes more time. Therefore, it is recommended to preheat fluids before pipetting in the MTP, which is yet another time‐consuming step of manual laboratory work. In the future, the replacement of PLA components in the cwMBR holder with aluminum components can further decrease the heating time.

Figure 3B displays the temperature in eight cwMBR chips and eight randomly distributed wells of a 96 well plate or a FlowerPlate in the related devices after 40 min of incubation. Therefore, the temperature deviations in the cwMBR are relatively low (Figure 3B1). A maximum temperature difference of 0.7°C can be observed. It is noticeable that the outer positions of the cwMBR platform are particularly affected, which can be explained by a more effective cooling from the colder ambient air at the beginning of the experiment. By using materials with a higher thermal conductivity like aluminum, this effect can be further reduced in future. In the conventional MBR (Figure 3B2), a maximal temperature difference of 0.6°C was observed, which is no significant difference to the cwMBR (*p* = 0.83). In contrast, significant temperature differences can be observed between the plate reader with 96‐well plate and the cwMBR (Figure 3B3, *p* = 0.015). The temperature difference of 3.2°C is within the same range observed in previous publications with edge effects in the MTP [22].

These experiments demonstrate the improved performance of the heating system in the cwMBR compared to established platforms. Both, temperature distribution as well as heating time, exceed the performance of conventional systems. MTP readers are often not constructed for heating cultivation media. Therefore, the cwMBR, with its small volume, short heating times, and homogenous temperature distribution can be a promising future alternative. Due to their small space requirements, the optical temperature sensors are one of the most suitable solutions for temperature measurement in the small volume of the cwMBR. However, the accuracy of these sensors, which is only 0.5 K, must be considered during the interpretation of the temperature profiles in the investigated microsystems. Even though the accuracy of the sensors needs improvement, the results clearly indicate a homogeneous temperature distribution in the cwMBR and short heating times.

### 3.3 Parallelized Spectrometric Light Detector

**FIGURE 4:**
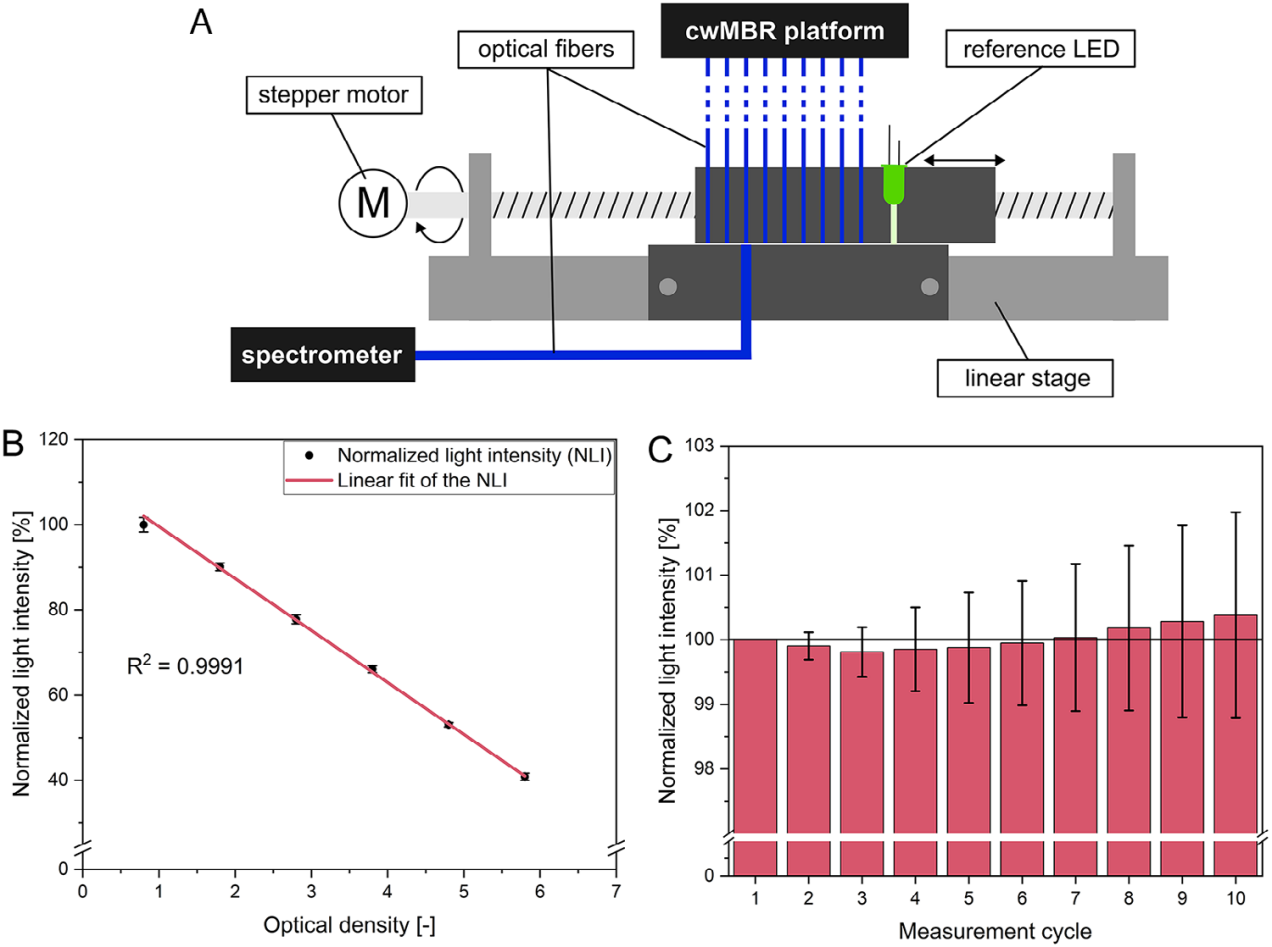
(A) Parallelized spectrometric light detector (PSLD): optical fibers (400 µm) conduct the transmitted light from the cwMBR chips to a linear stage. The linear stage with a stepper motor can align the fibers to a 1000 µm fiber connected to a spectrometer. If a cwMBR is illuminated by the LED as light source, the corresponding optical fiber is aligned and the transmitted light intensity is measured by the spectrometer. (B) Normalized light intensity (NLI) with linear fit measured by the PSLD at optical densities between 1 and 6 of a turbidity standard in the cwMBR. The first value was set to 100%. Each value represents the mean and the standard deviation from a triplicate. (C) NLI over ten cycles of measurement using the PSLD and empty cwMBR chips. The first value was set to 100%. Each value represents the mean and the standard deviation from a triplicate.

For application in phagograms but also for biomass growth or lysis characterization, online biomass measurement is essential in the cwMBR. Therefore, absorbance measurement is the most reliable and cost‐effective way of biomass measurement in the cwMBR. In previous setups, an LED was positioned over each cwMBR chip as a light source and either photoresistors or spectrometers were used as read‐out devices connected to the cwMBR bottom via optical fibers [28, 29]. Growing biomass in the cwMBR leads to a reduced transmitted light intensity in these setups, and the increasing absorbance can be calculated using the Beer‐Lambert law. Thereby, photoresistors are the more cost‐effective way for online measurement of the transmitted light intensity and, therefore, enable high parallelization. However, the resolution of photoresistors is limited compared to a spectrometer. Moreover, photoresistors are not able to measure the light intensity at a specific wavelength, which is important, for example, for absorbance measurement in colorimetric assays or future fluorescence measurements. Even though spectrometers can perform this task, connecting one spectrometer with a single port to each cwMBR would be associated with high costs for the devices. For these reasons, a cost‐effective parallelized PSLD was constructed (Figure 4A), which is able to measure the transmitted light intensity of numerous cwMBR chips at a specific wavelength using a single spectrometer.

The PSLD consists of a linear stage with spindle axle, a stepper motor, a spectrometer, optical fibers of different diameters, and mountings holding the fibers in position. Thin optical fibers (Ø 400 µm) are connected to each cwMBR, conducting the transmitted light to the PSLD. A thicker optical fiber (Ø 1000 µm) connects the spectrometer and the PSLD. For read‐out of a specific cwMBR chip, the linear stage aligns the chip‐corresponding fiber face‐to‐face to the fixed spectrometer fiber, so the transmitted light from the cwMBR chip can be conducted to the spectrometer. The linear stage is connected to a stepper motor for accurate movement of the fibers in 0.025 mm steps. The stepper motor is controlled by the same microcontroller, which controls the automated cwMBR platform. This allows measurement of the transmitted light intensity, when the cwMBR is illuminated by the measurement LED. For the alignment of the cwMBR fibers with the spectrometer fiber, a reference LED was implemented. An in‐house programmed Python script allows real‐time data monitoring for each cwMBR of the automated platform. The system can be simply extended for even higher numbers of parallel MBRs.

After the construction of the PSLD, it was characterized regarding the linearity of the sensor signal and the OD in the cwMBR chips (Figure 4B) as well as regarding the repeatability of the positioning (Figure 4C). According to the Beer‐Lambert law, the biomass concentration in the cwMBR should be linear to the absorbance and, therefore, the measured light intensity. To prove the linearity of the automated cwMBR and demonstrate the applicability of the PSLD, the cwMBR chips within the automated platform were filled with a turbidity standard with predefined optical densities. Afterwards, the light intensity was measured and normalized between the maximal light intensity of this experiment (100%) and a switched‐off LED (0%). The results indicate clear linearity (*R* ^2^ = 99.91%) between light intensity and OD, which was also expected under the Beer‐Lambert law (Figure 4B). The linearity was observed over the investigated OD range from 1 to 6, indicating the applicability of the sensor system within this OD range. Previous experiments [29] suggest ODs in *E. coli* based phagograms of up to approximately 4, making the PSLD suitable for *E. coli*‐based phagograms. Compared to the previously applied photoresistor‐based OD‐sensor [29], the linearity of the system was increased significantly (*R* ^2^ = 95.07%), indicating a more reliable sensor system. Furthermore, the resolution of the OD sensor system was increased using a spectrometer.

Besides the linearity of the sensors’ signals, repeatability of the LED and fiber positioning is crucial for accurate measurement. To achieve accurate positioning of the LED over the cwMBR chips as well as face‐to‐face positioning of the fibers in the PSLD, highly precise linear stages with spindle axles and stepper motors with a resolution of 1.8° per step were applied. These systems are normally applied for highly accurate 3D printers. Position control of the LED was achieved using a light barrier. To test if the PSLD measures a constant light intensity over several measurements, the average light intensity over nine empty cwMBR chips was measured over ten cycles. The first light intensity was set to 100%, and all other values show the deviation from the initial value (Figure 4C). Within ten measurement cycles, the NLI just slightly fluctuates within less than 0.4% from the initial value indicating good reproducibility over several measurement cycles. The highly precise spindle axle and stepper motor applied for the automated cwMBR platform fulfill their task. They allowing accurate OD measurement over a cultivation also within a parallelized setup. This setup is, therefore, perfectly suited to perform automated biomass measurement within automated phagograms in the cwMBR.

### 3.4 Application for Phagograms

**FIGURE 5:**
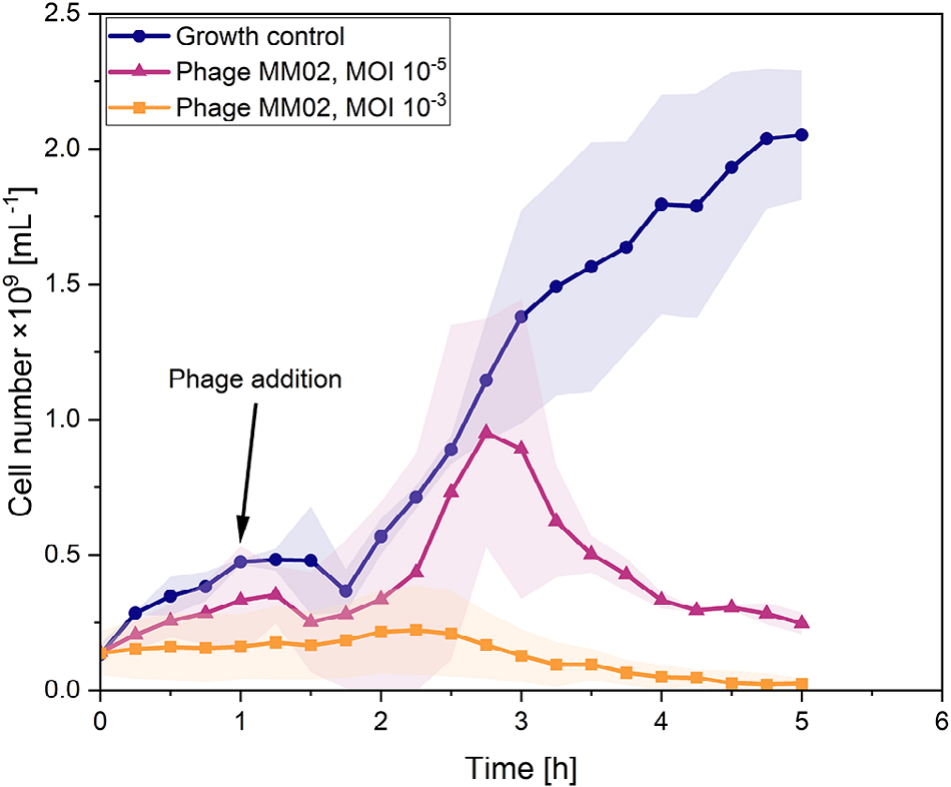
Cell number of *E. coli* during a phagogram in the automated cwMBR platform with phage MM02 (MOI of 10^−3^ and 10^−5^) at 37°C in complex medium (oscillation frequency of the cwMBR 70 Hz, amplitude 5%). The graphs show the mean and the standard deviation (lightly colored) from triplicates. Automated infection using the nanodispenser with the phages was performed at *t* = 1 h of incubation of the culture in the cwMBR without phages. The second nanodispenser was applied to compensate for evaporation by adding deionized water every 15 min beginning after 15 min of cultivation.

After the development and characterization of the automated cwMBR platform, it was applied to a model phagogram to demonstrate its applicability. *E. coli* and phage MM02 were used as a model as this model system was established in a previous publication [29]. It has already been shown that it is generally possible to perform phagograms using an unautomated version of the cwMBR platform with different phages and MOIs and that the model system is perfectly suited for detecting bacterial lysis. The MOI describes the ratio of phages to bacteria. However, these experiments had to be conducted manually by laboratory staff. The experiments were also limited by a less accurate sensor system and an inhomogeneous temperature distribution. Hence, the aim of this experiment was to demonstrate that the established phagograms from a previous publication [29] can also be performed in the automated cwMBR setup. Therefore, the cwMBR was filled with a preculture of *E. coli* at the beginning of the cultivation. After 1 h of biomass growth in the cwMBR, the phages were automatically added by the nanodispenser. To simulate different lysis behaviors, two different MOIs of 10^−3^ and 10^−5^ were added. For comparison, a growth control of *E. coli* without phages was cultivated. The results are displayed in Figure 5. After a lag phase of approximately 1.5 h, a distinct increase in cell number was observed for the growth control and the lower MOI of 10^−5^. In contrast, no clear increase in cell number was observed for the culture with an MOI of 10^−3^. In this culture, the cell concentration declines after remaining constant for approximately 2.5 h. At this point, phage lysis exceeds bacterial growth until the bacterial concentration is nearly zero at the end of the cultivation. After approximately 3 h, the cell number in the cultivation with an MOI of 10^−5^ also decreases, indicating a dominant influence of bacterial lysis by phages. The control culture continues to grow until the end of the experiment. Differences in the growth behavior before the addition of phages in the cwMBR chips with an MOI of 10^−5^ and the growth control can be explained by differences in the liquid level of the cwMBR chips. While the growth control was filled with 7 µL cultivation medium, the cwMBR chips were not completely filled for investigation of phage lysis in order to have space for the subsequent addition of phage. This leads to a shift in the resonance frequency of the droplet. Since the cwMBR chips were mixed with the resonance frequency of a 7 µL droplet, the deviating liquid level led to unfavorable mixing conditions and thus to reduced bacterial growth [15, 27]. In the future, this issue can be solved by the integration of a microchannel on the bottom of the cwMBR chip connected to a microfluidic pump for medium removal. The microchannel has already been integrated in a previous cwMBR setup [26]. In this setup, the cwMBR could be fully filled at the beginning of the experiment for optimal growth. The excess medium could be removed directly before phage addition.

The experiment clearly demonstrates the applicability of the automated cwMBR platform for phagograms and the ability to determine bacterial growth in the growth control. Furthermore, the nanodispenser was able to automatically add variable phage MOIs to the cwMBR resulting in different lysis behaviors. The characterization of the lysis kinetics is a key advantage of the phagogram applied in the cwMBR platform compared to established plaque assays. While plaque assays can only describe the extent to which a phage can lyse a specific bacterial strain, the lysis kinetics generated here provide important information about the speed of lysis within a shorter period of time. Plaque assays are usually performed over a period of several hours to a day, whereas preliminary results in the cwMBR can be observed in less than 4 h [31, 37–40]. Rapid and timely phagogram results are key factors for the success of phage therapy, especially for severely infected patients [41]. Furthermore, the phagogram in the cwMBR is automated resulting in reduced manual laboratory work and can, therefore, reduce the costs of the whole screening process. Moreover, the cwMBR platform can also be highly parallelized in a future cwMBR setup without the limitations of MTPs outlined above. These include unfavorable heating performance and temperature distributions as well as phage adhesion to the hydrophobic plastic material, as described in the literature [35]. In addition, many established highly parallelized plate readers used in clinical microbiology with multiple MTPs are limited in terms of online biomass determination and often only lead to endpoint determinations, which is a suboptimal gain of knowledge. In comparison, lab‐scale plate readers with online biomass determination, which have already been applied for various phage studies [37, 42, 43, 44, 45], are usually limited in terms of parallelizability. In contrast, the small space requirements of the cwMBR cavity enables high parallelization. The experiment also demonstrates the very good applicability of the automated cwMBR to compare the bacterial lysis at different MOIs. Since the clinical application of a maximum phage concentration is usually not possible, the determination of an optimal in vitro MOI is crucial for the success of phage therapy. MOIs that are too low can lead to an overall growth inhibition instead of bacterial lysis, which has been shown for various phages including MM02 [29, 42, 43, 46]. Therefore, this experiment clearly demonstrates the very good applicability of the cwMBR to accelerate the generation of phagograms in the future. Hence, increased parallelization of the cwMBR, as well as flexibilization of the nanodispenser, still needs to be performed to screen large phage libraries, phage cocktails, or highly promising phage‐antibiotic combinations in the future.

## 4 Concluding Remarks

The automation and parallelization of bioprocesses on the microscale is key to improve their performances, leading to higher efficiency, reduced costs, and improved reproducibility. Therefore, the development of novel MBRs for specific pharmaceutical applications such as phagograms is crucial in order to maximize the benefits of microsystems. The automated cwMBR platform is a system that perfectly combines automated bioprocesses with high parallelizability, data generation by optical sensors, active mixing, a minimal volume, and a hydrophilic glass surface, making the cwMBR perfectly suitable for various applications to solve bioprocesses challenges. The characterization of the mixing performance [15], the sensor integration [28], and the application of the cwMBR for phagograms [29] have already been demonstrated in previous publications. Since all previous experiments were performed in a non‐automated cwMBR platform, automation of the cwMBR platform was the next challenge, whose solution is presented here. Therefore, the cwMBR platform was extended by an automated nanodispenser on a linear stage, which is able to dispense liquids in the single‐digit nanoliter range for automated phage addition and to compensate for evaporation. For biomass determination, a highly parallelizable PSLD was developed, which can also determine the absorbance at different wavelengths in a highly parallelized cwMBR platform. The automated cwMBR platform is mainly heated by a heating foil, which leads to short heating times and a homogenous temperature distribution during the cultivation experiment. To validate the automated cwMBR platform for phagograms, a model phagogram was performed. This experiment demonstrates that automated experiments can be performed with reduced manual laboratory work and the recording of lysis kinetics in the highly parallelizable platform with a phage‐repellent hydrophilic glass surface.

To perform fully automated high‐throughput phagograms in the future, some modifications to the platform are necessary. These include the extension of the nanodispenser for further phage suspensions and a device for switching liquids. The switching device allows the LHS to switch flexibly between different phage solutions, water, detergents, or disinfectants in order to avoid cross‐contamination. Various disinfectants such as ethanol, peracetic acid, sodium hypochlorite, liquid alkaline chlorinated foam cleaner, and also UV light have phage‐inactivating properties [47]. Furthermore, high parallelization can significantly increase the number of parallel experiments. Therefore, the cwMBR cavities have to be parallelized in a second spatial direction and the distance between the cavities has to be reduced to significantly increase the number of parallel batches occupying a minimal surface area. In this case, a second spindle axle enables the nanodispensers to be moved in both directions. By integrating additional nanodispensers, the processing time can remain low even with high levels of parallelization. The PSLD remains well‐suited for a highly parallelized cwMBR platform. The fabrication of an aluminum cwMBR holder can further increase the temperature distribution and heating performance and thus further minimize the deviations between the cwMBR chips. With these optimizations, extended phagograms can be performed with large phage libraries, phage cocktails, or highly promising phage‐antibiotics combinations. In addition to phagograms, other applications such as biopharmaceutical assays and cell analysis are also possible to generate with such a cwMBR platform.

## Conflicts of Interest

The authors declare no conflicts of interest.

## Supporting information



Supporting information

## Data Availability

The data that support the findings of this study are available from the corresponding author upon reasonable request.
