# Supplementary material for: Automation of a Capillary‐Wave Microbioreactor Platform to Enhance Phage Sensitivity Screen Efficiency
Source: Eng Life Sci. 2025 Apr 14;25(4):e70021. doi: 10.1002/elsc.70021 (PMC11997256; doi:10.1002/elsc.70021)
Supplement: Supplementary file 1 — Supporting information [file ELSC-25-e70021-s001.docx]

Supplementary Material: Automation of a capillary-wave microbioreactor platform to enhance phage sensitivity screen efficiency

Kevin Viebrock (k.viebrock@tu-braunschweig.de)^a,c^, Ilka Knoke (i.knoke@tu-braunschweig.de) ^a,c^, Leon Huß (l.huss@tu-braunschweig.de) ^a,c^, Detlev Rasch (d.rasch@tu-braunschweig.de) ^a,c^, Sven Meinen (s.meinen@tu-braunschweig.de) ^b,c^, Andreas Dietzel (a.dietzel@tu-braunschweig.de) ^b,c^, Rainer Krull (r.krull@tu-braunschweig.de) ^a,c,^*

^a^ Institute of Biochemical Engineering, Technische Universität Braunschweig, Rebenring 56, 38106 Braunschweig, Germany;

^b^ Institute of Microtechnology, Technische Universität Braunschweig, Alte Salzdahlumer Str. 203, 38124 Braunschweig, Germany;

^c^ Center of Pharmaceutical Engineering, Technische Universität Braunschweig, Franz-Liszt-Str. 35a, 38106 Braunschweig, Germany

* Corresponding author

# S1 Arduino script for the automated cwMBR platform

This Arduino script describes the software of the Arduino Mega, which controls all devices of the automated cwMBR platform.

#include <Servo.h>

//Values for NEMA17 motor (PSLD)

const int BM_mot = 53; // pin1 for the biomass stepper motor

const int BM_dir = 52; // pin2 for the biomass stepper motor

const int BM_pos[] = {325,176,174,170,170,183,169,180,168}; //Positions of the optical fibers for biomass determination

//Values for NEMA23 motor (nanodispenser)

const int motdir = 12; // motor direction: low = carriage to the right; high = carriage to the left

const int motpin = 13; // motor pin for rotation through signal

const int LHSpin = 38; //pin connected to the trigger of the nanodispenser control unit

const int LEDpin = 44; //pin connected to the blue LED

const int GreenLED = A15; //pin connected to the reference LED in the PSLD

const int resis = A15;

const int OscillationPin = A0; //pin connected to the relays for interrupting the power supply of the oscillation

const int mbrnumber = 9; //number of cwMBR chips

const int FirstSlot = 22;

int NewActSlot = FirstSlot;

int motdelay = 3;

const int lhsdelay = 40; //IMPORTANT! Must match the length of droplet addition from NANODISPENSER

const int Oscillation_delay = 500;

const int droplet_delay = 500;

const int Spektro_delay = 2000;

int NotAus = 0;

const int NotAusSteps = 8750;

const int Messwerte_Foto = 100;

const int cavitySteps = 400;

const int between_cavity_steps = 742; //Distance between cavities. FIXED at 742 steps

const int LEDsteps = 495;

const int lastcwMBRsteps = 485 - LEDsteps;

const int openServo = 13.5;

long starttime = 0;

int delay_loop = 1;

int firstrun = 1; //Deactivates NANODISPENSER for 1st direct (t=0) BM measurement. 2 deactivates the function. Default: 1

//Variables for positioning NANODISPENSER A, LED and NANODISPENSER B over the reactor

const int LHSB_steps_begin = 410; // Steps that NANODISPENSER B needs to move from the start position at LS (firstpos = 0) to position itself above the first reactor

int Durchgange = 0; // Counts the number of loop iterations. Necessary for the timing of phage addition. Default: 0

const int Zugabe_Durchgang = 6; //Iteration in which phage addition takes place. At -1, NANODISPENSER B is never activated

Servo servo[mbrnumber];

const byte servoPins[] = {2,3,4,5,6,7,8,9,10}; // To the right: less

const byte servoInitPos [] = {117,54,90,62,65,86,59,104,118}; //To set the reactor lid positions

//Data type int can store up to a maximum of 32,767 milliseconds; data type long can store up to 24.8 days

const long evapdelay = 600000; //15 min: 900000 //10 min: 600000

const long start_delay = 120000; // 2 min: 120000

void setup() {

Serial.begin(9600);

pinMode(BM_mot, OUTPUT);

pinMode(BM_dir,OUTPUT);

pinMode(motdir, OUTPUT);

pinMode(motpin,OUTPUT);

pinMode(LHSpin, OUTPUT);

pinMode(OscillationPin, OUTPUT);

pinMode(GreenLED, OUTPUT);

pinMode(LEDpin, OUTPUT);

for (int s = 0; s < mbrnumber; s++){

servo[s].attach(servoPins[s]);

}

for (int s = 0; s < mbrnumber; s++){

servo[s].write(servoInitPos[s]);

}

digitalWrite(LEDpin, HIGH);

digitalWrite(motdir, LOW);

digitalWrite(BM_mot, LOW);

digitalWrite(OscillationPin, LOW);

digitalWrite(GreenLED, HIGH);

}

void loop() {

//movebackw();

//moveforw();

//Activate_GreenLED();

int activeLS = 1;

int pos = 0;

int LSsignal = SearchActiveLS();

int first_pos = 200;

if (delay_loop == 1){ // If loop that is executed only once at the start to correctly position the carriage for the rest of the experiment

delay(start_delay);

delay_loop++;

if ((LSsignal == 0) && (NewActSlot == FirstSlot)){ // Moves the carriage to the right until the first light barrier is triggered

while (activeLS >= 1){

NotAus ++;

NotAus = Motorbewegung(NotAus);

activeLS = digitalRead(NewActSlot);

}

}

first_pos = 0; // Sets the position where the first light barrier is triggered to 0. The code section: moves from the first light barrier position to the chosen start point 200 steps to the left

digitalWrite(motdir, HIGH); // motdir set to HIGH should change the motor direction to the left

while (first_pos != 200){ // Keeps motdir set to HIGH until first_pos is 200

Motorbewegung(NotAus); // first_pos is the first position of the carriage after code start

first_pos++;

}

digitalWrite(motdir, LOW); // Sets motor direction to the right

delay(1000);

}

starttime = millis(); // Time measurement: Start of reactor loop

NotAus = 0; // NotAus = 0 sets the zero point of the motor movement (200 steps to the left from the first light barrier)

int activembr = 1;

while (first_pos != 0){ // Moves from start position to the first light barrier position

Motorbewegung(NotAus);

first_pos--;

NotAus ++;

}

int mysteps = 0;

while (mysteps < LHSB_steps_begin){ // Motor movement to position NANODISPENSER B over the first reactor for the remainder of the addition loop

Motorbewegung(NotAus);

NotAus ++;

mysteps ++;

}

delay (500);

if (Durchgange != Zugabe_Durchgang){ // Procedure only for evaporation and biomass

while (activembr <= (mbrnumber+1)){ // +1 because NANODISPENSER A is always 1 reactor ahead of activembr. Loop needs to go one step further than BM

mysteps = 0;

while (mysteps < (between_cavity_steps) && activembr == 1){ // Motor movement of BM_LED over the first reactor, since NANODISPENSER B is not used in the loop

Motorbewegung(NotAus);

NotAus ++;

mysteps ++;

}

if (activembr <= mbrnumber){

Biomasse(activembr, Durchgange);

}

if (activembr > 1 && activembr <= (mbrnumber+1)){

Tropfenzugabe(activembr, firstrun, Durchgange);

}

if (activembr < (mbrnumber+1)){ // The carriage does not need to move to the next reactor at the end of the code

mysteps = 0;

while (mysteps < between_cavity_steps){

Motorbewegung(NotAus);

NotAus ++;

mysteps ++;

}

}

activembr ++;

}

}

else { // Phage addition after a fixed number of iterations (Zugabe_Durchgang)

while (activembr <= (mbrnumber+2)){

if (activembr <= mbrnumber){

TropfenzugabeBeta(activembr, Durchgange);

}

if (activembr > 1 && activembr <= (mbrnumber+1)){

Biomasse(activembr, Durchgange);

}

if (activembr > 2 && activembr <= (mbrnumber+2)){

Tropfenzugabe(activembr, firstrun, Durchgange);

}

if (activembr < (mbrnumber+2)){ // The carriage does not need to move to the next reactor at the end of the code

mysteps = 0;

while (mysteps < between_cavity_steps){

Motorbewegung(NotAus);

NotAus ++;

mysteps ++;

}

}

activembr ++;

}

}

delay(1000);

motdelay = 1;

digitalWrite(motdir, HIGH); // Change motor direction to the left to return to the starting position

while (NotAus > 0){ // Keep rotating the motor until it reaches position 0

NotAus --;

Motorbewegung(NotAus);

delay(1);

}

int BM_mot_pos = 0; // Move the NEMA17 spectrometer carriage back to the starting position

int BM_all_pos = 0;

for (int n = 0; n <= (mbrnumber); n++){

BM_all_pos = BM_all_pos + BM_pos[n];

}

digitalWrite(BM_dir, HIGH);

while (BM_mot_pos < BM_all_pos){

BM_mot_pos++;

BM_Motorbewegung();

delay(1);

}

digitalWrite(BM_dir, LOW);

digitalWrite(motdir, LOW);

motdelay = 3;

NotAus = 0;

long stoptime = millis(); // Interval time between each measurement

if((evapdelay - (stoptime - starttime)) < 0){

delay(1000);

}

else{

if (delay_loop == 1){ // Time interval considering the initial positioning at the first start of the code

delay(evapdelay - (stoptime - starttime) - start_delay);

}

else{

delay(evapdelay - (stoptime - starttime)); // Time interval for the rest of the measurements

}

}

firstrun ++;

Durchgange ++;

}

//////////////////////////////////////////////////////////////////////////////////

void movebackw(){

int backpos = 0;

while(backpos != 10000){

digitalWrite(motdir, HIGH);

NotAus = Motorbewegung(0);

}

}

//////////////////////////////////////////////////////////////////////////////////

void moveforw(){

int forwpos = 0;

while(forwpos != 10000){

digitalWrite(motdir, LOW);

NotAus = Motorbewegung(0);

}

}

//////////////////////////////////////////////////////////////////////////////////

void Activate_GreenLED(){

digitalWrite(GreenLED, HIGH);

delay(100000);

}

//////////////////////////////////////////////////////////////////////////////////

int SearchActiveLS(){

for (int i = FirstSlot; i <= (mbrnumber + FirstSlot - 1); i ++){

int signal = digitalRead(i);

if (signal <= 0){

return (i - FirstSlot + 1);

}

}

return 0;

}

//////////////////////////////////////////////////////////////////////////////////

// Water addition against evaporation using NANODISPENSER A

void Tropfenzugabe(int servonumber, int firstrun, int Durchgange){ //servonumber is used with activembr and firstrun with the firstrun variable

int number = 0;

if (firstrun > 1){

if (Durchgange != Zugabe_Durchgang){ // If loop exclusively for the evaporation cycle

digitalWrite(OscillationPin, HIGH);

delay(Oscillation_delay);

servo[servonumber-2].write(servoInitPos[servonumber-2]-openServo); // Open servos. -2 because NANODISPENSER A is 1 reactor behind the biomass in this loop

delay(500);

while (number < 20){ // Line determines how often the NANODISPENSER is triggered. Adjust based on evaporation rate and BioFluidix software settings

digitalWrite(LHSpin, HIGH);

delay (lhsdelay);

digitalWrite (LHSpin, LOW);

number ++;

}

delay(droplet_delay);

digitalWrite(OscillationPin, LOW);

servo[servonumber-2].write(servoInitPos[servonumber-2]);

}

else{ // Else loop for the one-time case of phage addition. Adjust evaporation amount to the dosage of NANODISPENSER B phage addition (function TropfenzugabeBeta)

digitalWrite(OscillationPin, HIGH);

delay(Oscillation_delay);

servo[servonumber-3].write(servoInitPos[servonumber-3]-openServo); // Open servos. -3 because NANODISPENSER A is 2 reactors behind NANODISPENSER B in this loop

delay(500);

if ((servonumber - 2) == 1 or (servonumber - 2) == 4 or (servonumber - 2) == 7){ // Growth control reactors. No phages --> Need normal amount of water against evaporation

while (number < 1){

digitalWrite(LHSpin, HIGH);

delay (lhsdelay);

digitalWrite (LHSpin, LOW);

number ++;

}

}

else if ((servonumber - 2) == 2 or (servonumber - 2) == 5 or (servonumber - 2) == 8){ // Reactors with low MOI. Need almost the same amount of water as without phages

while (number < 1){

digitalWrite(LHSpin, HIGH);

delay (lhsdelay);

digitalWrite (LHSpin, LOW);

number ++;

}

}

else {delay(1000);} // Reactors with higher MOI. Due to a lot of phage solution, no water is needed

delay(droplet_delay);

digitalWrite(OscillationPin, LOW);

servo[servonumber-3].write(servoInitPos[servonumber-3]);

}

}

else {delay(500);}

}

//////////////////////////////////////////////////////////////////////////////////

//Phage addition with NANODISPENSER B

void TropfenzugabeBeta(int servonumber, int Durchgange){ // servonumber is used with activembr and firstrun with the firstrun variable

int number = 0;

if (Durchgange != Zugabe_Durchgang){

delay(500);

}

else{ // Adjust evaporation amount to the dosage of NANODISPENSER B phage addition volume

digitalWrite(OscillationPin, HIGH);

delay(Oscillation_delay);

servo[servonumber-1].write(servoInitPos[servonumber-1]-openServo); // Open servos. -1 because NANODISPENSER B is always at activembr

delay(500);

if (servonumber == 1 or servonumber == 4 or servonumber == 7){ // Growth control reactors. No phages --> short delay. no manual addition.

delay(500);

}

else if (servonumber == 2 or servonumber == 5 or servonumber == 8){ // Reactors with low MOI (10-5). Only 1 shot of phages.

while (number < 1){

digitalWrite(LHSpin, HIGH);

delay (lhsdelay);

digitalWrite (LHSpin, LOW);

number ++;

}

delay(droplet_delay);

}

else { // Reactors with higher MOI (10-3). 100 shots of phages

while (number < 100){

digitalWrite(LHSpin, HIGH);

delay (lhsdelay);

digitalWrite (LHSpin, LOW);

number ++;

}

delay(droplet_delay);

}

digitalWrite(OscillationPin, LOW);

servo[servonumber-1].write(servoInitPos[servonumber-1]);

}

}

//////////////////////////////////////////////////////////////////////////////////

void Biomasse(int LSsignal, int Durchgange){ // LSsignal represents the number of the active MBR (1 to 9)

if (Durchgange != Zugabe_Durchgang){

digitalWrite(OscillationPin, HIGH); // Turn off mixing

servo[LSsignal-1].write(servoInitPos[LSsignal-1]-openServo); // Open servo over which the LED stands

Activate_Spectrometer(LSsignal, 0); // Measure biomass with the LED off

Activate_Spectrometer(LSsignal, 1); // Measure biomass with the LED on

Activate_Spectrometer(LSsignal, 1);

servo[LSsignal-1].write(servoInitPos[LSsignal-1]); // Close servo

digitalWrite(OscillationPin, LOW); // Turn on mixing

delay(Oscillation_delay);

}

else{

digitalWrite(OscillationPin, HIGH); // Turn off mixing

servo[LSsignal-2].write(servoInitPos[LSsignal-2]-openServo); // Open servo over which the LED stands

Activate_Spectrometer((LSsignal-1), 0); // Measure biomass with the LED off

Activate_Spectrometer((LSsignal-1), 1); // Measure biomass with the LED on

Activate_Spectrometer((LSsignal-1), 1);

servo[LSsignal-2].write(servoInitPos[LSsignal-2]); // Close servo

digitalWrite(OscillationPin, LOW); // Turn on mixing

delay(Oscillation_delay);

}

}

//////////////////////////////////////////////////////////////////////////////////

void Activate_Spectrometer(int LSsignal, int Zero){

int curr_pos = 0;

delay(100);

if (Zero == 0){

digitalWrite(BM_dir, LOW);

while(curr_pos < BM_pos[LSsignal-1]){

BM_Motorbewegung();

curr_pos++;

}

delay(10);

Serial.print("cwMBR_Zero_");

Serial.println(LSsignal);

delay(Spektro_delay);

}

digitalWrite(LEDpin, LOW);

delay(100);

Serial.print("cwMBR_Val_");

Serial.println(LSsignal);

delay(Spektro_delay);

digitalWrite(LEDpin, HIGH);

delay(100);

}

//////////////////////////////////////////////////////////////////////////////////

void BM_Motorbewegung(){

digitalWrite(BM_mot, HIGH);

delay(motdelay);

digitalWrite(BM_mot, LOW);

delay(motdelay);

}

int Motorbewegung(long stop){

if (stop >= NotAusSteps){

Serial.println("Motorfehler. Neustart.");

//while(1){};

delay(10000);

int helpstop = 0;

motdelay = 1;

digitalWrite(motdir, HIGH);

while (helpstop < (NotAusSteps + 500)){

digitalWrite(motpin, HIGH);

delay(motdelay);

digitalWrite(motpin, LOW);

delay(motdelay);

helpstop ++;

}

motdelay = 3;

digitalWrite(motdir, LOW);

return 0;

}

else {

digitalWrite(motpin, HIGH);

delay(motdelay);

digitalWrite(motpin, LOW);

delay(motdelay);

return stop;

}

}

# S2 Arduino script for the temperature control of the incubation chamber

This Arduino script describes the software of the Arduino Uno, which controls the temperature in the incubation box by adjusting the rotation speed of the fans.

// Sketch to control the ambient temperature of the cwMBR using

// Sensor addressing via Arduino Uno

// Temperature comparison with the target temperature

// Control of the fans based on the comparison

// Circulating cooler heats constantly to 40 °C

// Including the libraries

#include <OneWire.h>

#include <DallasTemperature.h>

#define ONE_WIRE_BUS_1 8 // DS18B20 sensor at digital pin 8 (HIGH and LOW)

OneWire oneWire(ONE_WIRE_BUS_1); // Setup OneWire instance to communicate with any OneWire device

// Passing the OneWire reference to communicate with the sensor

DallasTemperature sensors(&oneWire);

// Integer for Serial Monitor output

int val = 0;

int maxRPM = 255; // Maximum PWM output for Arduino Uno

int currTime = 0;

int heating = 1;

// Integer for warning signal LED

const int warnLED = 7;

// Integer for safety check

int sensorCount;

// Definition of variables

float offset = 1.2; // Offset of the temperature sensor

float TTar = 37; // Target temperature adjusted by the offset

float LB = TTar - 1;      // First lower permissible temperature limit

float LBend = TTar - 4;   // Lowest permissible temperature limit

float UB = TTar + 1;      // First upper permissible temperature limit

float UBend = TTar + 4;    // Highest permissible temperature limit

// Definition of the pin

const int PWMOutput = 5; // PWM fan wire at analog pin 5 (to control fan speeds between 0 and 255)

void setup() {

 Serial.begin(9600); // Establish serial communication to transfer measurement data to Processing

 pinMode(warnLED, OUTPUT);

 digitalWrite(warnLED, LOW);

 pinMode(PWMOutput, OUTPUT); // Define PWMOutput pin as output

 analogWrite(PWMOutput, 0); // Turn off ventilation at the beginning

 sensors.begin();

 sensorCount = sensors.getDS18Count(); // Read the number of connected temperature sensors (for safety)

}

void loop() {

  if (sensorCount < 1) { // Check if any sensor signal is present

    Serial.println("No temperature sensor found.");

  }

 sensors.requestTemperatures(); // Command to read temperatures

 currTime = currTime + 1;

 float currTemp = (sensors.getTempCByIndex(0)+offset); // Assign the current temperature to currTemp

 if (heating == 0){

  Preheat();

  heating ++;

 }

// Only one sensor considered (Index 0)

// Check if the current temperature matches the target temperature

// Adjust the fan speed based on the temperature difference between target and current temperature

 if (currTemp < LBend) { // If the current temperature is below the lowest permissible temperature limit

  analogWrite(PWMOutput, 255); // the fans should run at maximum speed

  val = 255*100/maxRPM;

  digitalWrite(warnLED, HIGH);

 }

 else if ((currTemp < LB) && (currTemp >= LBend)){  // If the current temperature is below the first lower temperature limit

   analogWrite(PWMOutput, 220); // the fans should run at a medium speed

   val = 200*100/maxRPM;

   digitalWrite(warnLED, HIGH);

 }

 else if ((currTemp < TTar) && (currTemp >= LB)){   // If the current temperature is between target temperature and lower temperature limit

   analogWrite(PWMOutput, 200); // the fans should run at a medium speed

   val = 150*100/maxRPM;

   digitalWrite(warnLED, LOW);

 }

 else if (currTemp == TTar){ // If the current temperature equals the target temperature

   analogWrite(PWMOutput, 120); // the fans should run at a low speed

   val = 120*100/maxRPM;

   digitalWrite(warnLED, LOW);

 }

 else if ((currTemp > TTar) && (currTemp <= UB)){   // If the current temperature is between the target temperature and the upper temperature limit

   analogWrite(PWMOutput, 50); // the fans should run at a low speed

   val = 70*100/maxRPM;

   digitalWrite(warnLED, LOW);

 }

 else if ((currTemp > UB) && (currTemp <= UBend)){ // If the current temperature is above the upper temperature limit but below the highest permissible temperature limit

   analogWrite(PWMOutput, 20); // the fans should run at a very low speed

   val = 20*100/maxRPM;

   digitalWrite(warnLED, HIGH);

 }

 else if (currTemp > UBend){ // If the current temperature is above the highest permissible temperature limit

   analogWrite(PWMOutput, 10); // the fans should run slowly to ensure some circulation

   val = 10*100/maxRPM;

   digitalWrite(warnLED, HIGH);

 }

 delay(1000); // 1-second interval

 Serial.println(currTemp);

 Serial.println(currTime);

}

void Preheat(){

  float currTemp = (sensors.getTempCByIndex(0)+offset);

  while (currTemp < (TTar - 0.5)){

    analogWrite(PWMOutput, 255);

    currTemp = (sensors.getTempCByIndex(0)+offset);

    Serial.println(currTemp);

    Serial.println(currTime);

    currTime ++;

    delay(1000); // 1-second interval

  }

}

# S3 Python script for the online data visualization via PSLD

This Python script can visualize data measured by the spectrometer in the PSLD in a graphical user interface and stores the data in a text file.

import multiprocessing

import tkinter as tk

from tkinter import filedialog

import pandas as pd

import serial

import time

from serial.tools import list_ports

import keyboard

import sys

import matplotlib.animation as animation

from matplotlib import style

from datetime import datetime

import matplotlib.pyplot as plt

import seabreeze

import os

import numpy as np

import math

from matplotlib.animation import FuncAnimation

seabreeze.use('cseabreeze')

from seabreeze.spectrometers import list_devices

import seabreeze.spectrometers as sb

import tkinter as tk

from tkinter import messagebox

ani = None # Define ani as a global variable

def script1():

txt_name_str = "Spektrometer_Data.txt"

txt_name = txt_name_str.split("\\")[-1]

com_var_default = "Kein COM ausgewählt"

def create_txt():

if os.path.exists(txt_name):

os.remove(txt_name)

open(txt_name, "a").close()

create_txt()

def update_txt(data1, data2, data3):

with open(txt_name, "a") as file:

file.write(f"{data1},{data2},{data3}\n")

def save_data_to_file(file_path, file_extension, txt_name):

if file_extension == "txt":

# Save as a text file

with open(txt_name, 'r') as source_file:

with open(file_path, 'w') as destination_file:

for line in source_file:

destination_file.write(line)

print("Die Daten wurden als .txt gespeichert.")

elif file_extension == "xlsx":

# Save as an Excel file

df = pd.read_csv(txt_name, sep=',')

df.to_excel(file_path, index=False)

print("Die Daten wurden als .xlsx gespeichert.")

else:

print("Ungültiger Dateityp.")

os.remove(txt_name)

def spectrometer_data(duration_ms, vals, mean_intensity, cwMBR):

path_name_str = "Spectrometer_Data.txt"

lowerB = 480

upperB = 490

compensation_times = [2700,1750,3400,2600,3400,2100,2400,2400,3600]

mycwMBR = int(str(cwMBR[-1]))

integration_time = compensation_times[mycwMBR-1]

devices = list_devices()

if len(devices) != 0:

spec = sb.Spectrometer(devices[0])

else:

print("Kein Spektrometer angeschlossen.")

return

start_time = time.time()

duration_sec = duration_ms / 1000.0 # Convert duration to seconds

while (time.time() - start_time) < duration_sec:

spec.integration_time_micros(integration_time)

x = np.around(spec.wavelengths(), 2, out=None)

y = np.around(spec.intensities(), 2, out=None)

num_y = 0

denom = 0

for j in range(len(x)):

if x[j] >= lowerB and x[j] <= upperB:

num_y = num_y + y[j]

denom += 1

if denom > 0:

num_y = num_y / denom

if vals == 1:

mean_intensity = num_y

else:

mean_intensity = ((mean_intensity * (vals - 1)) + num_y) / (vals)

vals += 1

curr_time = time.strftime("%H:%M:%S", time.localtime())

update_txt(curr_time, mean_intensity, cwMBR)

spec.close()

def start_reading_data(ser, file_path, file_extension):

start_time = time.strftime("%H:%M:%S", time.localtime())

tk.messagebox.showinfo(title=None,

message="Um das Skript zu beenden, bitte 'q' drücken. \n \nDie Daten werden dann am gewählten Speicherort abgelegt.")

print("\n" + "Program started at " + start_time + "\n")

while True:

if keyboard.is_pressed("q"):

save_data_to_file(file_path, file_extension, txt_name)

ser.close()

sys.exit()

elif ser.in_waiting:

prev_data = 0

data = ser.readline().decode('utf-8').strip()

if prev_data != data:

spectrometer_data(duration_ms=1000, vals=1, mean_intensity=0, cwMBR=data)

prev_data = data

time.sleep(0.001) # Wait for 1 second

def start_button_clicked():

# Open the common file dialog to select the save location and file name

file_path = filedialog.asksaveasfilename(defaultextension=".txt",

filetypes=[("Text Files", "*.txt"), ("Excel Files", "*.xlsx")])

# Check if a file path was selected

if file_path:

# Extract the file extension

file_extension = file_path.split(".")[-1]

else:

print("Es wurde kein Dateipfad ausgewählt.")

return

selected_com = com_var.get()

if selected_com == com_var_default:

print("Bitte einen vorhandenen COM-Port auswählen.")

return

try:

ser = serial.Serial(selected_com, 9600)

start_button.config(state=tk.DISABLED)

window.withdraw()

start_reading_data(ser, file_path, file_extension)

except serial.SerialException:

print("Es konnte keine Verbindung mit Arduino hergestellt werden.")

def update_start_button_state(*args):

selected_com = com_var.get()

if selected_com == com_var_default:

start_button.config(state=tk.DISABLED)

else:

start_button.config(state=tk.NORMAL)

# Create the main window

window = tk.Tk()

window.title("Serielle Kommunikation")

# Set the window dimensions

window_width = 400

window_height = 200

window.geometry(f"{window_width}x{window_height}")

# Get the available COM ports

com_ports = [port.device for port in list_ports.comports()]

default = [com_var_default]

com_ports = default + com_ports

# Create a label for the COM port selection

com_label = tk.Label(window, text="Select COM Port:")

com_label.pack()

# Create a dropdown menu for COM port selection

com_var = tk.StringVar(window)

com_var.set(com_ports[0]) # Set default value

com_dropdown = tk.OptionMenu(window, com_var, *com_ports)

com_dropdown.pack()

# Add a trace to detect changes in the COM port selection

com_var.trace("w", update_start_button_state)

# Create a frame for button placement

button_frame = tk.Frame(window)

button_frame.pack(pady=10)

# Create start button

start_button = tk.Button(button_frame, text="Start", command=start_button_clicked, state=tk.DISABLED)

start_button.pack(side=tk.LEFT, padx=5)

# Center the button frame

button_frame.place(relx=0.5, rely=0.5, anchor=tk.CENTER)

# Anchor the button frame below the dropdown menu

button_frame.pack(anchor=tk.CENTER, pady=10)

# Run the main event loop

window.mainloop()

def script2():

filename = "Spektrometer_Data.txt"

def create_txt(txt_name):

if os.path.exists(txt_name):

os.remove(txt_name)

open(txt_name, "a").close()

create_txt("Spektro_Mittelwert")

def find_best_mean(xs):

mean_all = sum(xs)/3

mean_pair1 = (xs[0] + xs[1]) / 2

mean_pair2 = (xs[0] + xs[2]) / 2

mean_pair3 = (xs[1] + xs[2]) / 2

if mean_pair1 > mean_pair2:

max_mean = mean_pair1

else:

max_mean = mean_pair2

if mean_pair3 > max_mean:

max_mean = mean_pair3

if (max_mean * 100/ mean_all) <= 95 or (max_mean * 100/ mean_all) >= 105:

return max_mean

else:

return mean_all

def plot_animation(filename):

style.use('fivethirtyeight')

axs = [3,3]

fig, axs = plt.subplots(3, 3, sharex=True)

def x_axis_val(x, reference_time):

time_obj = datetime.strptime(x, '%H:%M:%S')

time_diff = (time_obj - reference_time).total_seconds() / 3600

return round(time_diff, 4)

def animate(i, filename):

graph_data = open(filename, 'r').read()

lines = graph_data.split('\n')

ys = []

best_xs = [[0] for _ in range(10)]

best_ys = [[0] for _ in range(10)]

color_pattern = [['red', 'blue', 'green'],

['orange', 'purple', 'cyan'],

['pink', 'brown', 'gray']]

reference_time = None

x = 0

best_mean = 0

cwMBR_number = 0

for line_num, line in enumerate(lines):

if len(line) > 1:

if line_num % 1 == 0:

x, y, z = line.split(',')

if reference_time is None:

reference_time = datetime.strptime(x, '%H:%M:%S')

x = x_axis_val(x, reference_time)

if "cwMBR" in z:

if "cwMBR_Zero_" in z:

zparts = z.split("cwMBR_Zero_")

elif "cwMBR_Val_" in z:

zparts = z.split("cwMBR_Val_")

else:

print("Error")

if len(zparts) == 2:

cwMBR_number = int(zparts[1])

else:

cwMBR_number = 10

if "cwMBR_Zero_" in z:

y_zero = float(y)

elif "cwMBR_Val_" in z:

y_val = float(y)

ys.append(y_val)

else:

print("Error.")

else:

cwMBR_number = 10

if len(ys) == 3:

best_mean = find_best_mean(ys)

##if (best_ys[cwMBR_number - 1][-1] * 0.75) < best_mean or (best_ys[cwMBR_number - 1][-1] * 1.25) > best_mean:

best_mean = best_mean - y_zero

best_xs[cwMBR_number - 1].append(x)

best_ys[cwMBR_number - 1].append(best_mean)

##else:

##print("Zu große Abweichung um " + x + " Uhr in cwMBR" + cwMBR_number + ".")

ys = []

#if best_mean > 60000:

#print("Spektrometer ist vermutlich übersättigt.")

for i in range(9):

row_index = i // 3

col_index = i % 3

color = color_pattern[row_index][col_index]

axs[row_index, col_index].set_ylabel("absorbance [-]")

axs[row_index, col_index].set_xlabel("time [h]")

axs[row_index, col_index].set_title(f"cwMBR {i + 1}")

# Check if there is data to plot for this y_number

if best_ys[i]: # If ys[i] is not empty

axs[row_index, col_index].plot(best_xs[i], best_ys[i], color = color)

plt.tight_layout()

ni = animation.FuncAnimation(fig, animate, fargs=(filename,), interval=2000, save_count=10)

plt.show()

plot_animation(filename)

if __name__ == '__main__':

devices = list_devices()

if len(devices) == 0:

tk.messagebox.showinfo(title=None,

message="Es wurde kein Spektrometer angeschlossen.")

# Create two process objects for each script

process1 = multiprocessing.Process(target=script1)

process2 = multiprocessing.Process(target=script2)

# Start both processes

process1.start()

process2.start()

# Wait for both processes to finish

process1.join()

process2.join()
